# PsCor413pm2, a Plasma Membrane-Localized, Cold-Regulated Protein from *Phlox subulata*, Confers Low Temperature Tolerance in *Arabidopsis*

**DOI:** 10.3390/ijms19092579

**Published:** 2018-08-30

**Authors:** Aimin Zhou, Enhui Liu, He Li, Yang Li, Shuang Feng, Shufang Gong, Jingang Wang

**Affiliations:** 1 College of Horticulture and Landscape Architecture, Northeast Agricultural University, Harbin 150030, China; aiminzhou@neau.edu.cn (A.Z.); liuenhui1024@aliyun.com (E.L.); 18845798249@163.com (H.L.); liyang44@ibcas.ac.cn (Y.L.); shufanggong@neau.edu.cn (S.G.); 2 Key Laboratory of Saline-Alkali Vegetation Ecology Restoration in Oil Field (SAVER), Ministry of Education, Alkali Soil Natural Environmental Science Center (ASNESC), Northeast Forestry University, Harbin 150040, China; shuangfeng1986@aliyun.com

**Keywords:** cold stress, cold-regulated gene, *Phlox subulata*, PsCor413pm2, transgenic *Arabidopsis*

## Abstract

Low temperature stress adversely affects plant growth and development. Isolation and characterization of cold response genes from cold-tolerant plants help to understand the mechanism underlying low temperature tolerance. In this study, *PsCor413pm2*, a cold-regulated (COR) gene isolated from *Phlox subulata*, was transferred to *Arabidopsis* plants to investigate its function. Real-time quantitative PCR analysis revealed that *PsCor413pm2* expression was induced by cold. Subcellular localization revealed that the PsCor413pm2-green fluorescent protein (GFP) fusion protein localized to the plasma membrane in tobacco and *Arabidopsis* plants. Furthermore, overexpression of *PsCor413pm2* in *Arabidopsis* plants enhanced tolerance to low temperature stress. Transgenic *Arabidopsis* roots had more influx of Ca^2+^ after a cold shock than wild-type plants, as shown using non-invasive micro-test technology (NMT). Moreover, the transcription abundance of five *COR* and two C-repeat (CRT) binding factor (CBF) genes in transgenic *Arabidopsis* plants was higher than that in the wild-type plants under cold stress. Taken together, our results suggest that overexpression of *PsCor413pm2* enhances low temperature tolerance in *Arabidopsis* plants by affecting Ca^2+^ flux and the expression of stress-related *COR* and *CBF* genes.

## 1. Introduction

Low temperatures adversely affect plant growth, development, and crop productivity [1]. During the process of long-term adaptive evolution, some temperate plant species have evolved a series of physiological and molecular mechanisms to acclimate to low temperatures. Among these mechanisms, cold signaling pathways dependent on C-repeat binding factors (CBFs) are the best-understood regulatory pathways involved in cold acclimation [2,3].

Cold stress is sensed by membrane proteins, leading to a cytosolic Ca^2+^ signal. Ca^2+^-binding proteins may mediate the Ca^2+^ signal to activate a series of downstream transcription factors, such as inducer of CBF expression 1 (ICE1), which is the upstream regulator of CBF. CBF proteins can bind to C-repeat (CRT) cis-elements and activate transcription of the downstream cold-regulated (COR) genes to increase cold tolerance [1,4,5,6]. Studies have shown that expression of *COR* genes is positively correlated with cold tolerance in plants [7,8,9,10]. *COR* genes, such as *Cor6.6*, *Cor15*, *Cor47*, *Cor78*, and *Cor413* have been identified in *Arabidopsis* plants [11,12,13]. Among these genes, *AtCor15* and *AtCor78* are the most characterized. Reports have shown that AtCor15 is a chloroplast stromal protein that protects chloroplast membranes during freezing stress [14,15,16]. Further, *AtCor78*, also known as *AtRD29A* (responsive to dehydration, RD), is involved in the response to cold, dehydration, and salt stressors [17,18,19]. 

The Cor413 family of proteins has been divided into two distinct groups, including Cor413-plasma membrane (Cor413pm) and Cor413-inner membrane (Cor413im) proteins [13]. Studies have shown that *Arabidopsis* AtCor413im localizes to the inner membrane of chloroplasts and may stabilize the chloroplast membrane under cold stress [20,21]. However, Cor413pm localization to the plasma membrane and its functions in response to cold stress have not been reported. Recently, we characterized the functions of *PsCor413im1* from *Phlox subulata*, which are cold-tolerant plants. Overexpression of *PsCor413im1* enhanced cold tolerance in *Arabidopsis* plants [22]. Here, we identified PsCor413pm2 as another member of the Cor413 family and conducted a preliminary evaluation of its functions using transgenic *Arabidopsis* plants. Subcellular localization of PsCor413pm2 was performed using GFP as a marker. Using non-invasive micro-test technology (NMT), we then measured the effects of *PsCor413pm2* overexpression on Ca^2+^ flux in *Arabidopsis* roots under cold shock. Our study helps to further characterize the function of Cor413 proteins.

## 2. Results

### 2.1. Sequence Analysis of PsCor413pm2

**Figure 1:**
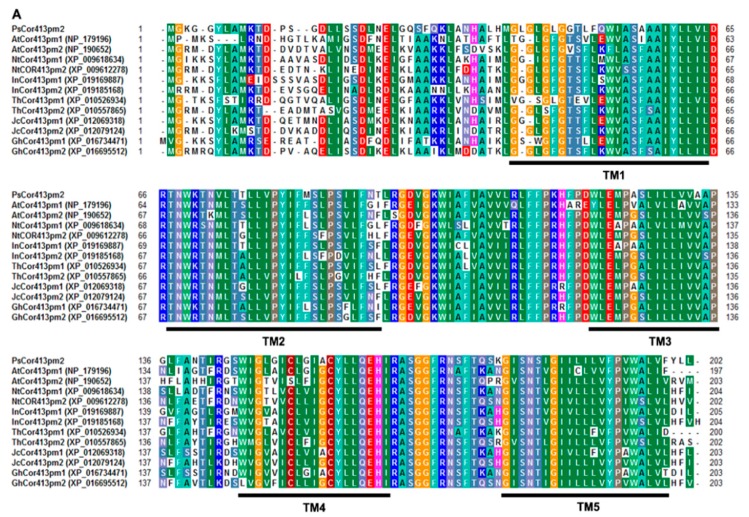

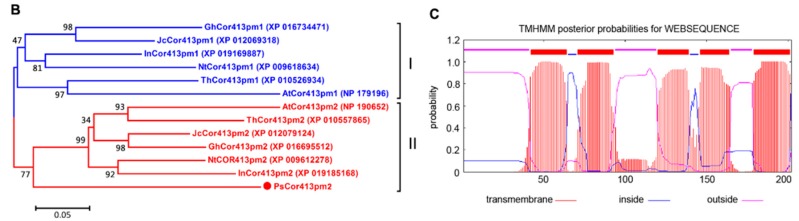
Sequence analysis of PsCor413pm2. Amino acid sequence alignment (**A**) and phylogenetic tree (**B**) of PsCor413pm2 with Cor413pm proteins from *Arabidopsis thaliana* (GenBank accession numbers: NP_179196 and NP_190652), *Nicotiana tomentosiformis* (XP_009618634 and XP_009612278), *Ipomoea nil* (XP_019169887 and XP_019185168), *Tarenaya hassleriana* (XP_010526934 and XP_010557865), *Jatropha curcas* (XP_012069318 and XP_012079124), and *Gossypium hirsutum* (XP_016734471 and XP_016695512) are shown. Colored backgrounds indicate identical residues. The black box bars indicate five putative transmembrane (TM) domains. (**C**) Putative transmembrane domains of PsCor413pm2 are shown.

**Figure 2:**
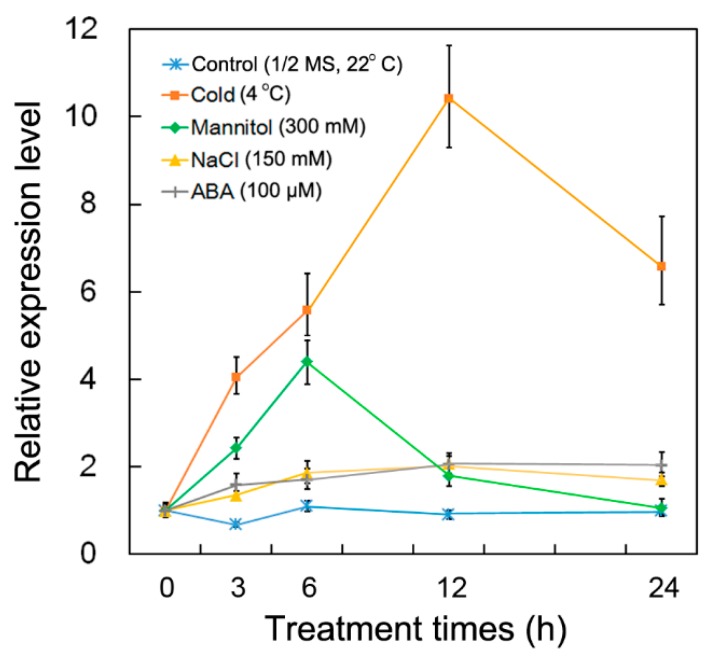
Expression of *PsCor413pm2* under multiple stresses. Two-week-old *Phlox subulata* seedlings were treated with cold (4 °C), mannitol (300 mM), NaCl (150 mM), and abscisic acid (ABA) (100 μM) for 0, 3, 6, 12, and 24 h. The *PsActin* gene was used as an internal control, and the transcript level in the untreated seedlings was set as 1.0. Error bars show the standard deviation (SD) of the values from three replicates.

The full-length *PsCor413pm2* cDNA contained a 609-bp open reading frame (ORF), which was predicted to encode a protein of 202 amino acids with a molecular mass of 22.07 kDa. Multiple sequence alignment and phylogenetic analyses revealed that PsCor413pm2 is highly homologous to two members of the Cor413pm protein family from other plant species (>77.11% identity), including *Arabidopsis thaliana*, *Nicotiana tomentosiformis*, *Ipomoea nil*, *Tarenaya hassleriana*, *Jatropha curcas*, and *Gossypium hirsutum* (Figure 1A,B). PsCor413pm2 was predicted to have five transmembrane domains using the TMHMM algorithm (Figure 1A,C). qPCR analysis showed that *PsCor413pm2* expression was obviously induced by cold (4 °C) and mannitol (300 mM) stress and slightly by NaCl (150 mM) and abscisic acid (ABA) (100 μM) (Figure 2). We add it, please confirm.

### 2.2. Subcellular Localization of PsCor413pm2

**Figure 3:**
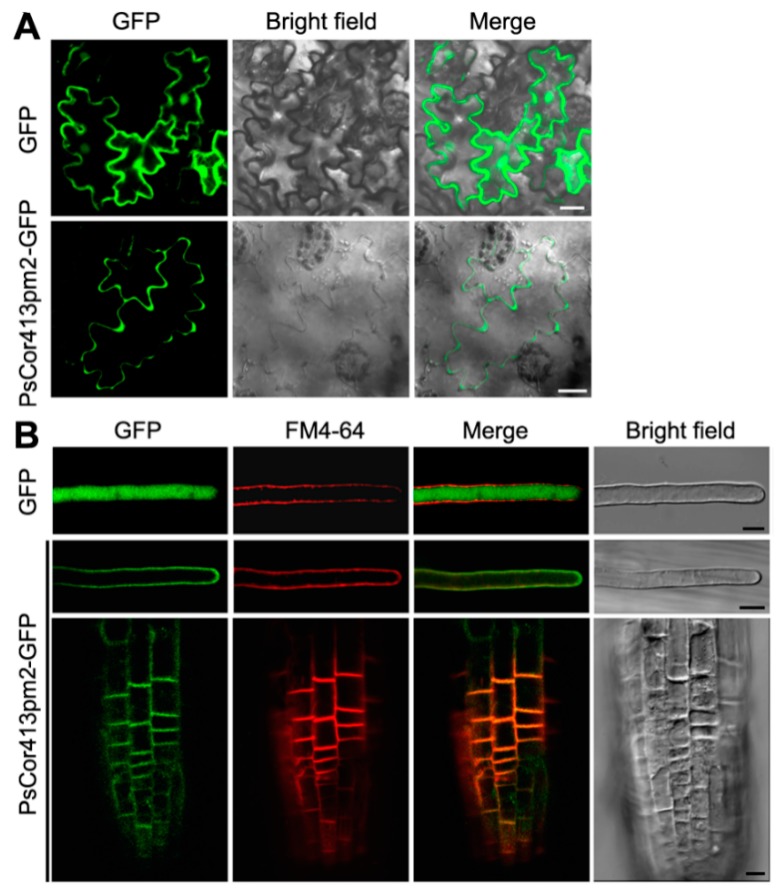
Subcellular localization of PsCor413pm2. (**A**) Confocal images of tobacco leaf epidermal cells transiently expressing GFP or PsCor413pm2-GFP are shown. (**B**) Confocal images of *Arabidopsis* root and root hair cells stably expressing GFP or PsCor413pm2-GFP incubated for 1 min with 4 μM FM4-64 are shown. GFP fluorescence is green, and FM4-64 is red. Merge pictures were created by merging the GFP and FM4-64 fluorescent images. Scale bars = 10 μm.

To confirm whether PsCor413pm2 is a membrane protein, localization of PsCor413pm2 in plant cells was examined using GFP as a fusion protein marker. In tobacco leaf epidermis cells transiently expressing PsCor413pm2-GFP, GFP signals were localized to the cell periphery, which is similar to the plasma membrane (Figure 3A). In *Arabidopsis* root and root hair cells stably expressing PsCor413pm2-GFP, the GFP signals were also observed in the cell periphery and co-localized with the plasma membrane marker dye FM4-64 (after staining for 1 min) (Figure 3B). However, when GFP alone was used as a control, it localized to the cytoplasm in tobacco leaf epidermis and *Arabidopsis* root hair cells (Figure 3A,B). These results suggest that PsCor413pm2-GFP localized to the plasma membrane.

### 2.3. Overexpression of PsCor413pm2 in Arabidopsis Enhances Tolerance to Low Temperature

**Figure 4:**
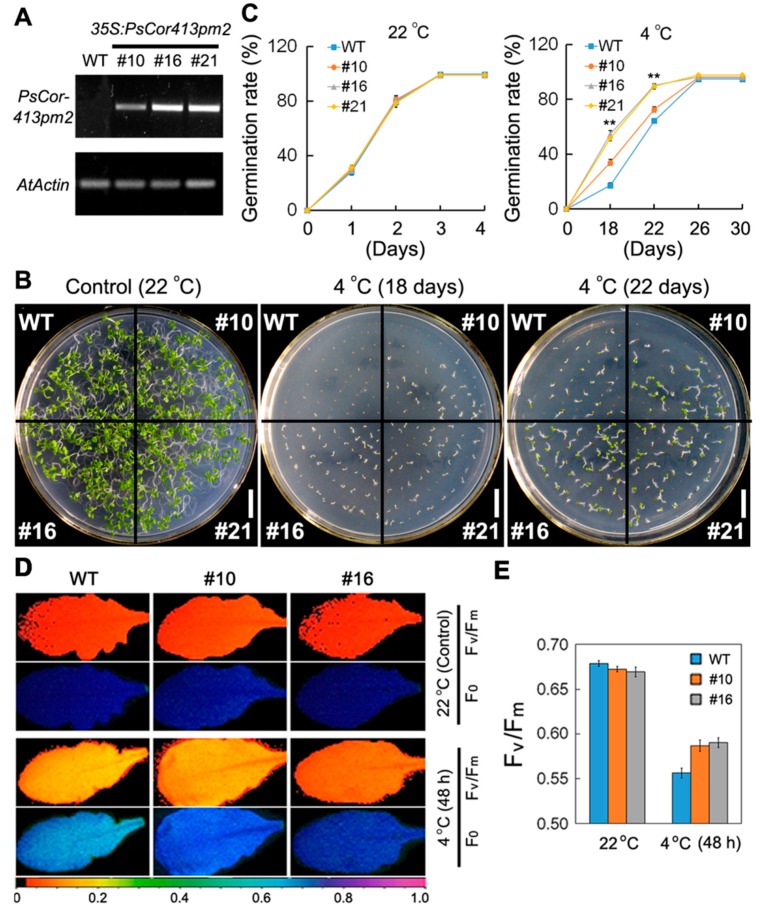
Phenotypes of transgenic *Arabidopsis* and wild type (WT) seedlings under low temperature stress. (**A**) Semi-quantitative PCR analyses of *PsCor413pm2* expression in WT and three transgenic *Arabidopsis* lines (#10, #16, and #21) overexpressing *PsCor413pm2*. (**B**,**C**) Comparison of germination rates in the WT and three transgenic lines on 1/2 strength Murashige and Skoog (MS) medium under normal (22 °C) and cold (4 °C) stress. Maximal photochemical efficiency (Fv/Fm) of transgenic *Arabidopsis* and WT leaves under cold stress. F_0_ and Fv/Fm images (**D**) and their quantification analysis (**E**) of leaves of WT and transgenic lines (#10 and #16) under normal (22 °C) and cold (4 °C, 48 h) stress. The pseudocolored bar depicted at the bottom of the panel ranges from 0 (black) to 1.0 (purple). Asterisks indicate significant differences between WT and transgenic lines (** *p* < 0.01; Student’s *t* test). Error bars show standard error (SE) of the values from three replicates. Scale bars = 1 cm.

**Figure 5:**
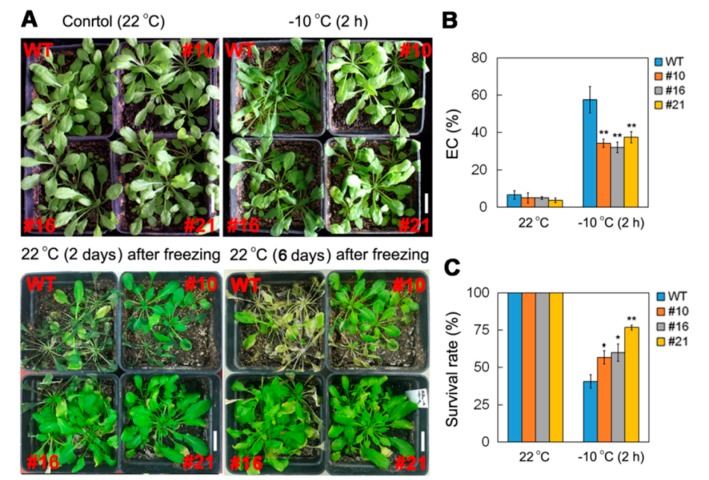
Comparison of phenotype (**A**), electrical conductivity (EC) (**B**), and survival rate (**C**) of WT and transgenic plants grown in soil under normal (22 °C) and freezing (−10 °C, 2 h) stress. Asterisks indicate significant differences between WT and transgenic lines (* *p* < 0.05; ** *p* < 0.01; Student’s *t* test). Error bars show standard error (SE) of the values from three replicates. Scale bars = 2 cm.

Transgenic *Arabidopsis* plants overexpressing *PsCor413pm2* driven by a CaMV35S promoter were generated (Figure 4A) to evaluate its possible role in the plant response to multiple stresses. First, we compared the germination rate of seeds from the transgenic lines and wild type (WT) plants on 1/2 strength Murashige and Skoog (MS) medium under cold (4 °C) stress. At normal temperatures (22 °C), germination rates in the transgenic seeds were similar to those of WT seeds. However, under cold (4 °C) stress conditions, germination rates of the transgenic seeds were significantly higher than those of WT seeds after 18 and 22 days of cultivation (Figure 4B,C). Moreover, we compared the maximum photochemical efficiency (Fv/Fm) of leaves of transgenic lines and WT plants growing in soil under normal and cold (4 °C) stress (Figure 4D). As shown in Figure 4E, cold (4 °C, 48 h) treatment resulted in marked decreases in Fv/Fm in both the WT and transgenic plants, but the Fv/Fm of the transgenic plants (#10 and #16) upon stress treatment was higher than that of the WT plants. Further, we compared electrical conductivity (EC) and survival rate of transgenic lines and WT plants growing in soil under normal and freezing (−10 °C) stress. The EC of transgenic plants was significantly lower than that of WT plants after freezing (−10 °C) treatment for 2 h (Figure 5A,B). The EC results indicated less damage from the freezing treatment in transgenic plant leaves than in WT leaves. Furthermore, the survival rate of transgenic plants was higher than that of WT after freezing (−10 °C) treatment (Figure 5A,C). These results suggest that overexpression of *PsCor413pm2* improves tolerance to low temperature stress in *Arabidopsis* plants. 

In addition, we compared seedling growth in the transgenic lines and WT plants under osmotic stress. On 1/2 strength MS supplemented with mannitol, germination rate (with 250 mM mannitol), primary root length, and fresh weight (with 175 and 200 mM mannitol) of the transgenic seedlings were significantly greater than those of WT plants (Supplementary Figure S1).

### 2.4. Overexpression of PsCor413pm2 in Arabidopsis Affects Ca^2+^ Flux and Stress-Related Genes Expression

**Figure 6:**
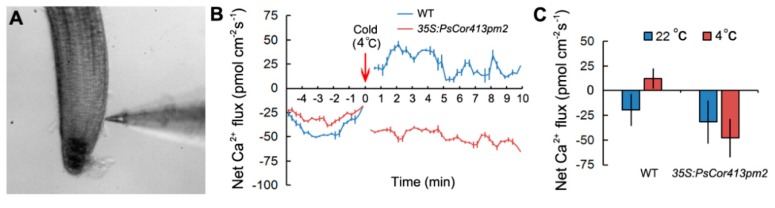
Effect of cold shock on net Ca^2+^ flux in the root elongation zone of transgenic *Arabidopsis* and wild type (WT) seedlings. (**A**) The root zones of *Arabidopsis* seedlings as measured using non-invasive micro-test technology (NMT). (**B**) Ca^2+^ kinetics recorded after a cold (4 °C) solution was added (at red arrow) to the chamber are shown. Prior to the cold shock, Ca^2+^ fluxes in roots were examined for approximately 5 min at room temperature (22 °C). (**C**) The mean Ca^2+^ flux rate in the root elongation zone of transgenic *Arabidopsis* plants (#10) overexpressing *PsCor413pm2*, as well as WT plants during the period of cold shock is shown. Error bars show the SE of the values from six replicates.

The initial plant response to cold was confirmed to be an influx of Ca^2+^ as a signaling molecule into the cytosol through the plasma membrane [1]. Net Ca^2+^ flux in response to cold shock in seedling roots from transgenic *Arabidopsis* and WT plants was monitored using NMT (Figure 6A). At normal temperatures (22 °C), there was a significant influx of extracellular Ca^2+^, with a minus peak in transgenic *Arabidopsis* and WT roots. Upon cold (4 °C) shock, WT roots exhibited a slight Ca^2+^ efflux, while transgenic *Arabidopsis* roots exhibited a greater influx of Ca^2+^ (Figure 6B). The mean Ca^2+^ influx in untreated transgenic *Arabidopsis* roots was slightly higher than that in WT plants. However, there were significant differences in the mean Ca^2+^ flux attributable to cold shock between transgenic *Arabidopsis* and WT plants (Figure 6C). These results suggest that overexpression of *PsCor413pm2* affects Ca^2+^ flux in *Arabidopsis* roots under cold shock.

**Figure 7:**
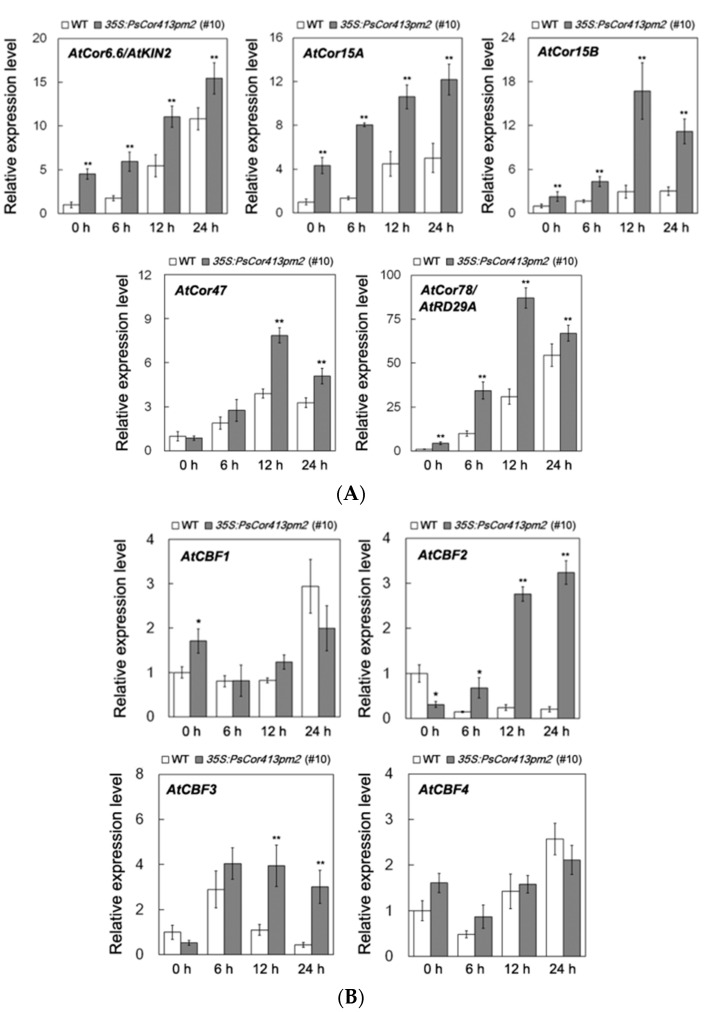
Expression of stress-associated genes in transgenic *Arabidopsis* and wild type (WT) seedlings under cold stress. Two-week-old transgenic *Arabidopsis* line (#10) overexpressing *PsCor413pm2* and WT seedlings were treated at 4 °C for the indicated time periods. Expression of the *AtCor6.6/AtKIN2*, *AtCor15A*, *AtCor15B*, *AtCor47*, *AtCor78/AtRD29* (**A**) and *AtCBF1* to *AtCBF4* (**B**) genes was investigated using qPCR. The *AtActin2* gene was used as an internal control, and the transcript level in the untreated WT seedlings was set as 1.0. Asterisks indicate significant differences between WT and transgenic lines (* *p* < 0.05; ** *p* < 0.01; student’s *t* test). Error bars show the SD of the values from three replicates.

Further, the expression of five *COR* genes (*AtCor6.6*/*AtKIN2*, *AtCor15A*, *AtCor15B*, *AtCor47*, and *AtCor78*/*AtRD29*) in transgenic *Arabidopsis* and WT plants was investigated under conditions of cold stress. Under normal and cold (4 °C) conditions, the transcript abundance of these five *COR* genes in transgenic *Arabidopsis* plants was evidently higher than in the WT plants (Figure 7A and Supplementary Figure S2A). The expression of many *COR* genes was regulated by CBF transcription factors. Further, we compared the expression of *AtCBFs* in transgenic *Arabidopsis* and WT plants under normal and cold (4 °C) stress. Under cold (4 °C) conditions, *AtCBF2* and *AtCBF3* expression from the *AtCBF* gene family was evidently higher than in the WT plants (Figure 7B and Supplementary Figure S2B). These results suggest that overexpression of *PsCor413pm2* affects expression of two *CBF* and five *COR* genes in *Arabidopsis* plants under cold stress.

## 3. Discussion

The CBF pathway activation is an important plant response pathway to low temperature stress. Plants likely sense low temperatures through plasma membrane proteins such as chilling-tolerance-divergence 1 (COLD1), which might induce a cytosolic Ca^2+^ signal and activate protein kinases [23]. Protein kinases are assumed to activate downstream transcription factors such as calmodulin-binding transcription activators (CAMTAs) and ICE1 through sumoylation and phosphorylation. ICE1-activated CBF regulates the expression of *COR* genes, which confer low temperature tolerance in plants [1,4,5,6]. *Cor413* is a member of the *COR* gene family, and it is divided into two groups, *Cor413pm* and *Cor413im*. In our study, the presence of a homolog of *Cor413pm2* was revealed in the genome of *P. subulata*. The sequence analysis showed that it might be a conserved membrane protein with five transmembrane domains (Figure 1). qPCR analysis showed that *PsCor413pm2* expression was obviously induced by cold and mannitol stress (Figure 2). Similarly, the *AtCor413pm2* expression in *Arabidopsis* was also induced by cold and mannitol stress (data from *Arabidopsis* eFP Browser). Subcellular localization of GFP as a fusion marker showed that PsCor413pm2-GFP was localized to the plasma membrane in *Arabidopsis* (Figure 3), indicating that the fusion of GFP did not affect the normal localization of PsCor413pm2. These results suggest that PsCor413pm2 is a cold-regulated protein located on the plasma membrane. Furthermore, seed germination, leaf photochemical efficiency, electrical conductivity, and survival rate analysis showed that *PsCor413pm2* transgenic *Arabidopsis* are more tolerant to low temperatures than WT (Figure 4 and Figure 5). In addition, our study showed that the transcript abundance of five *AtCOR* (*AtCor6.6/AtKIN2*, *AtCor15A*, *AtCor15B*, *AtCor47*, and *AtCor78/AtRD29*) and two *AtCBF* (*AtCBF2* and *AtCBF3*) genes from *PsCor413pm2* transgenic *Arabidopsis* was significantly higher than that of WT plants under cold stress (Figure 7 and Supplementary Figure S2). These results suggest that overexpression of *PsCor413pm2* in *Arabidopsis* may confer tolerance to low temperatures by influencing the expression of *COR* and *CBF* genes. NMT analysis revealed a greater Ca^2+^ influx in transgenic *Arabidopsis* roots under cold shock (Figure 6). Under cold stress, an increase in cytosolic Ca^2+^ as a signaling molecule activates downstream *COR* gene expression [24]. Therefore, the influx of Ca^2+^ may cause an increase in *COR* gene expression in transgenic *Arabidopsis*. However, how the overexpression of *PsCor413pm2* affects the flux of Ca^2+^ is unclear. We speculate that there are two possibilities. Subcellular localization showed that PsCor413pm2 is a plasma membrane protein (Figure 3), and it is known that the plasma membrane is the primary site of chilling and freezing injury. Therefore, the first proposed hypothesis is that PsCor413pm2 may be a plasma membrane protein associated with Ca^2+^ signaling. Similarly, COLD1, a cold-related protein localized on the plasma membrane in Japonica rice, also affects the flux of Ca^2+^ in the root under cold shock. Further, researchers have demonstrated that COLD1, as a regulator of G-protein signaling, confers cold tolerance by modulating Ca^2+^ signaling in rice [25,26]. Early studies predicted that Cor413 proteins are G protein-coupled receptors [13]. The second hypothesis is that PsCor413pm2 is a membrane-stabilizing protein. As an integral membrane protein, PsCor413pm2 could play a structural role by stabilizing the plasma membrane lipid bilayer. The stability of the plasma membrane contributes to the function of plasma membrane proteins, such as Ca^2+^-flux regulating related proteins. The expression of *AtCBF2* and *AtCBF3* in transgenic *Arabidopsis* may be affected by the change of Ca^2+^ flux caused by *PsCor413pm2* overexpression.

In addition, *PsCor413pm2* overexpression enhances tolerance to osmotic stress in *Arabidopsis* (Supplementary Figure S1). Under normal conditions, the expression of *AtCor78* in transgenic plants was significantly higher than that in WT plants (Figure 7 and Supplementary Figure S2A). *AtCor78* is also known as *AtRD29A*, which is involved in plant responses to dehydration stress [27]. Thus, overexpression of *PsCor413pm2* enhances osmotic tolerance in *Arabidopsis* plants and may be related to the enhanced expression of *AtCor78/AtRD29A* under normal conditions. 

Taken together, our results suggest that overexpression of *PsCor413pm2* enhances low temperature tolerance in *Arabidopsis* plants by affecting Ca^2+^ flux and the expression of stress-related *COR* and *CBF* genes. Nevertheless, we will continue to study these mechanisms, as they should be further evaluated and verified.

## 4. Materials and Methods

### 4.1. Identification of PsCor413pm2 and Sequence Analysis

Based on sequence similarity, the *PsCor413pm2* gene (GenBank accession number: KT337405.1) was identified using transcriptome sequencing data from cold-treated *P. subulata*. The amino acid sequences of PsCor413pm2 and its homologs were aligned using ClustalW. The transmembrane domains in PsCor413pm2 were predicted using the TMHMM algorithm [28], and the phylogenetic tree was constructed via the neighbor-joining method using the molecular evolutionary genetics analysis (MEGA) 4.1 software [29] with 1000 bootstrap replicates. The bootstrap scores <50% were deleted.

### 4.2. Plant Material and Growth Conditions

*P. subulata* plants were grown under controlled greenhouse conditions with 70–80% relative humidity, 14 h of light, and an average temperature of 22 °C. All *Arabidopsis thaliana* plants used in this study belonged to the Columbia-0 ecotype. The seeds were surface sterilized and stratified at 4 °C for 2 days in the dark. The seedlings were then grown on 1/2 strength MS medium (3% sucrose, 1% agar, pH 5.8) under a 12 h light/12 h dark photoperiod (100 μmol m^−2^ s^−1^ light intensity) at 22 °C.

For abiotic stress treatments, 2-week-old *P. subulata* or *Arabidopsis* seedlings were exposed to 4 °C, 100 μM ABA, 150 mM NaCl, or 300 mM mannitol. At least 10 seedlings from each treatment group were harvested and pooled at different time points (0, 3, 6, 12, or 24 h after treatment), frozen immediately in liquid nitrogen, and stored at −80 °C for RNA preparation.

### 4.3. RNA Extraction and Real-Time Quantitative PCR (qPCR) Analyses

Total RNA was extracted using a RNeasy mini kit (Qiagen, Valencia, CA, USA) according to the manufacturer’s instructions. First-strand cDNA was synthesized from 1 µg total RNA with the M-MLV RTase cDNA synthesis kit (TaKaRa, Shiga, Japan). Real-time quantitative PCR analysis was performed using the SYBR green mix (Agilent Technologies, Palo Alto, CA, USA) in an optical 96-well plate on a Mx3000P system (Agilent). Three biological repeats and three technical repeats were performed for qPCR analysis. The primers used in this study are shown in Supplementary Table S1.

### 4.4. Vector Construction and Plant Transformation

To create the pBI121-PsCor413pm2 construct, the ORF of *PsCor413pm2* was amplified via PCR and cloned at the XbaI and SacI sites of the pBI121 vector. To construct the GFP fusion genes, the ORF of *PsCor413pm2*, without the stop codon, was amplified using PCR and cloned at the XbaI and KpnI sites of the pBI121-GFP vector. The accuracy of the above constructs was confirmed by sequencing, and the specific primers used in this study are shown in Supplementary Table S1.

The constructs were transformed into the *Agrobacterium tumefaciens* strain EHA105 for plant transformation. Transient expression in tobacco leaves was performed according to a previously described procedure [22]. *Arabidopsis* plants were transformed using the floral dip method [30]; the transgenic plants were then selected on 1/2 strength MS medium containing 30 μg mL^−1^ kanamycin. Expression of *PsCor413pm2* in the transgenic lines was assessed via semi-quantitative reverse transcription PCR analyses using the T3 generation.

### 4.5. Confocal Laser Scanning Microscopy

The tobacco leaf epidermis was peeled to make a temporary squash and then visualized using confocal laser scanning microscopy (CLSM; Nikon, A1, Tokyo, Japan). Four-day-old *Arabidopsis* seedlings grown on vertical 1/2 strength MS agar plates were incubated in 1 mL liquid 1/2 strength MS medium (0.5% sucrose, pH 5.8) containing 4 μM FM4-64 (Invitrogen, Carlsbad, CA, USA) for 1 min at room temperature (22 °C). Roots of transgenic *Arabidopsis* seedlings were washed twice with liquid 1/2 strength MS medium immediately before visualizing via CLSM. GFP signals were detected using a 500–530 nm emission filter. FM4-64 signals were detected using a 620–680 nm emission filter.

### 4.6. Low Temperature and Osmosis Tolerance Assay

*Arabidopsis* seeds were treated at 4 °C for 2 days and then grown on 1/2 strength MS medium at different temperatures (22 °C or 4 °C) or 1/2 strength MS medium supplemented with mannitol (0, 175, 200 mM, or 250 mM) before measuring seed germination rate, seedling root length, and fresh weight. The experiment was replicated three times.

*Arabidopsis* plants grown in soil for 20 days were treated with 4 °C for 48 h or −10 °C for 2 h, then the leaves of 2 cm diameter were used for maximal Fv/Fm and relative EC analysis. Fv/Fm was measured using an Imaging-PAM Chlorophyll Fluorometer (M-series, Heinz Walz GmbH, Frankfurt am Main, Germany) as described previously [31]. The percent relative EC was calculated as EC% = Post freezing EC/Post autoclaving EC × 100. These experiments were replicated three times with ten plants per treatment. After the −10 °C treatment, the plants were transferred to light (12 h light/12 h dark photoperiod) at 22 °C. The survival rates of the plants were scored visually after 2 weeks. The experiment was repeated three times and the average values were calculated. For each experiment, 5 pots from WT and three transgenic lines were used, with 4–5 seedlings per pot.

### 4.7. Net Ca^2+^ Flux Measurements

Net Ca^2+^ flux was measured using NMT (NMT100 Series, Younger USA LLC, Amherst, MA, USA) as described previously [25,32]. Seven-day-old seedling root segments were immobilized in measuring solution (0.1 mM KCl, 0.1 mM CaCl_2_, 0.1 mM MgCl_2_, 0.5 mM NaCl, 0.3 mM 2-(N-morpholino) ethanesulfonic acid (MES), and 0.2 mM Na_2_SO_4_, pH 6.0) to measure Ca^2+^ flux. For sudden cold-shock treatment, transient Ca^2+^ flux kinetics in response to cold (4 °C) were measured in seedling root tips. After measuring for 5–6 min at room temperature, ice-cold test buffer (4 °C) was quickly added to the container around the roots; Ca^2+^ flux was simultaneously measured for a further 10 min. Six biological repeats were performed for each analysis.

### 4.8. Statistical Analyses

The data were analyzed via one-way analysis of variance using SPSS software (Version 19.0, SPSS Inc., Chicago, IL, USA), and statistically significant differences were calculated based on the student’s *t-*test, with *p* < 0.05 (*) and *p* < 0.01 (**) as the thresholds for significance.

## Supplementary Materials

Supplementary materials can be found at http://www.mdpi.com/1422-0067/19/9/2579/s1. Figure S1: Phenotypes of transgenic Arabidopsis and wild type (WT) seedlings under osmotic Stress; Figure S2: Expression of stress-associated genes in transgenic Arabidopsis and wild type (WT) seedlings under cold stress; Table S1: List of primers used in this study.
