# Supplementary material for: PsCor413pm2, a Plasma Membrane-Localized, Cold-Regulated Protein from Phlox subulata, Confers Low Temperature Tolerance in Arabidopsis
Source: Int J Mol Sci. 2018 Aug 30;19(9):2579. doi: 10.3390/ijms19092579 (PMC6164191; doi:10.3390/ijms19092579)
Supplement: Supplementary file 1 [file ijms-19-02579-s001.pdf]

Supplementary Information

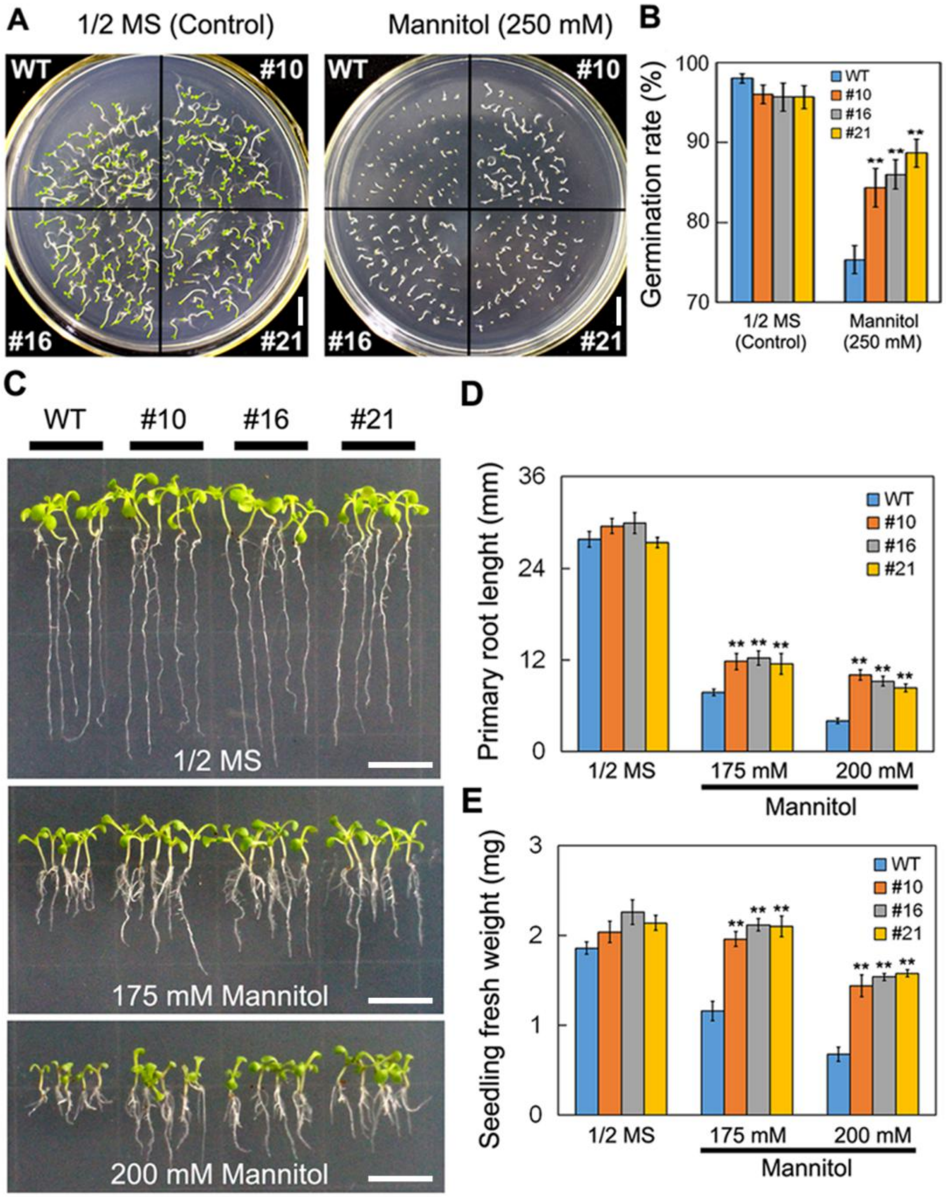

Figure S1. Phenotypes of transgenic *Arabidopsis* and wild type (WT) seedlings under osmotic stress. (**A**, **B**) Comparison of germination rates in the WT and three transgenic lines under normal (1/2 MS) and osmotic stresses (250 mM mannitol). Seedling growth (**C**), root length (**D**), and fresh weight (**E**) of WT and three transgenic lines on 1/2 MS medium supplemented with mannitol (0, 175, and 200 mM). Asterisks indicate significant differences between WT and transgenic lines (**$P < 0.01$; student's t test). Error bars show the standard error (SE) of the values from three replicates. Scale bars = 1 cm.

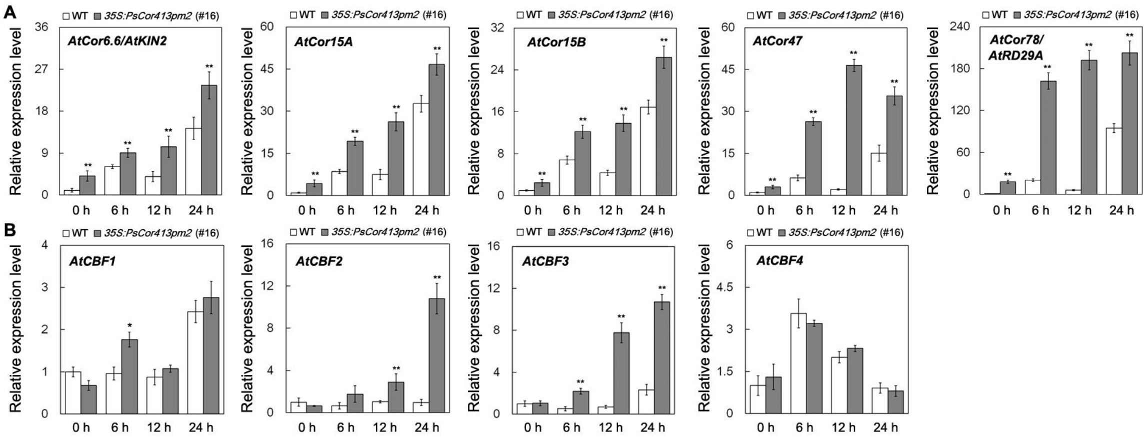

Figure S2. Expression of stress-associated genes in transgenic *Arabidopsis* and wild type (WT) seedlings under cold stress. Two-week-old transgenic *Arabidopsis* line (#16) overexpressing *PsCor413pm2* and WT seedlings were treated at 4 °C for the indicated time periods. Expression of the *AtCor6.6/AtKIN2*, *AtCor15A*, *AtCor15B*, *AtCor47*, *AtCor78/AtRD29* (**A**) and *AtCBF1* to *AtCBF4* (**B**) genes was investigated using qPCR. The *AtActin2* gene was used as an internal control, and the transcript level in the untreated WT seedlings was set as 1.0. Asterisks indicate significant differences between WT and transgenic lines (*$P < 0.05$; **$P < 0.01$; student's t test). Error bars show the standard deviation (SD) of the values from three replicates.

Table S1. List of primers used in this study.

Primer name	Primer sequence (5'→3')	Purpose
PsCor413pm2-qF	CACGAGGAACCGCAATACAG	qPCR
PsCor413pm2-qR	GAAGCAGCGACACAAAAGGT	qPCR
PsActin-qF	TTGGATTCTGGTGATGGTGT	qPCR
PsActin-qR	TCCACCGATGAACTGCTCTT	qPCR
AtCBF1-qF (At4g25490)	ACTTCGCTGACTCGGCTTGG	qPCR
AtCBF1-qR	ACGCACCTTCGCTCTGTTCC	qPCR
AtCBF2-qF (At4g25470)	TCAACCTGTGCCAAGGAAAT	qPCR
AtCBF2-qR	TAGCCTCCACCAAGGTCTCC	qPCR
AtCBF3-qF (At4g25480)	GCTATTTACACGGCGGAACA	qPCR
AtCBF3-qR	GCCAACAAACTCGGCATCTC	qPCR
AtCBF4-qF (At5g51990)	CGGAGGAGCAGAATGGTGGT	qPCR
AtCBF4-qR	CGTCAAAGTCGTTATGATTCCAG	qPCR
AtCor6.6/AtKIN2-qF (At5g15970)	GCAACAGGCGGGAAAGAGTA	qPCR
AtCor6.6/AtKIN2-qR	CGGATCGCTACTTGTTCAGGC	qPCR
AtCor15A-qF (At2g42540)	TCAGTTCGTCGTCGTTTCTCAA	qPCR
AtCor15A-qR	CACCATCTGCTAATGCCTCTTT	qPCR
AtCor15B-qF (At2g42530)	ATGGCGATGTCTTTATCAGGAG	qPCR
AtCor15B-qR	CGAGGATGTTGCCGTCACTT	qPCR
AtCor47-qF (At1g20440)	CGAGCGATGAAGAAGGTGAG	qPCR
AtCor47-qR	TGCTGTCTTGTCGTGGTGTC	qPCR
AtCor78/AtRD29A-qF (At5g52310)	CGTTTGCTCCAAGTGGTGAT	qPCR
AtCor78/AtRD29A-qR	GGCGAATCCTTACCGAGAACA	qPCR
AtActin2-qF (At3g18780)	GGTAACATTGTGCTCAGTGGTGG	qPCR
AtActin2-qR	AACGACCTTAATCTTCATGCTGC	qPCR
AtActin1-F (AT2G37620)	GAAAATGGCTGATGGTGAAG	RT-PCR
AtActin1-R	CTCATAGATAGGAACAGTGTGGC	RT-PCR
PsCor413pm2(XbaI)-F	TCTAGAATGGGGAAAGGCGGTTACT	Cloning and subcellular localization
PsCor413pm2(SacI)-R	GAGCTCCTAGAGGAGGTAGAATACCA	Cloning
PsCor413pm2(KpnI)-R	GGTACCAAGAGGAGGTAGAATACCAAAG	Subcellular localization
